# Association between the risk perception of contracting COVID-19 and sociodemographic characteristics in a Peruvian population

**DOI:** 10.12688/f1000research.138838.2

**Published:** 2023-10-30

**Authors:** Jhon Alex Zeladita-Huaman, David Esteban-Espinoza, Michelle Lozada-Urbano, Eduardo Franco Chalco, Marcelo Fernandes Costa, Henry Castillo-Parra

**Affiliations:** 1 Nursing Academic Department, Universidad Nacional Mayor de San Marcos, Lima District, Lima Region, Peru; 2 Departamento de Ciencias, Universidad Ricardo Palma, Santiago de Surco, Lima, Peru, Peru; 3 South American Center fo Education and Research in Public Health, Universidad Norbert Wiener, Lima District, Lima Region, Peru; 4 Research Department, Universidad Maria Auxiliadora, Lima District, Lima Region, Peru; 5 Departamento de Psicologia Experimental, Universidade de Sao Paulo, São Paulo, State of São Paulo, Brazil; 6 Facultad de Ciencias de la Salud, Universidad Maria Auxiliadora, Lima District, Lima Region, Peru

**Keywords:** Covid-19, Perception, Disease Susceptibility, Health Belief Model

## Abstract

**Background:** The perception of risk regarding coronavirus disease 2019 (COVID-19) has been widely researched due to its association with the adoption of preventive measures. In addition, since the onset of vaccination, it has been reported that the population perceives a lower risk of getting infected. However, few studies have analyzed the factors associated with risk perception in low- and middle-income countries. The aim of this study was to determine the association between the risk perception of contracting COVID-19 and sociodemographic characteristics in Peruvian population.

**Methods:** An analytical and cross-sectional study was conducted in four cities in Peru from October to December, 2021. The sample consisted of 821 individuals aged 18 years and older. A virtual questionnaire was used to collect sociodemographic data and assess the risk perception of contracting coronavirus based on the Health Belief Model. The process of back-translation, expert judgment, and reliability analysis using split-half correlation was conducted. Student’s t-tests, analysis of variance with post hoc Tukey’s test, and Spearman’s correlation were employed.

**Results:** Of the participants, 53.71% were women and 73.3% had a higher education level, 45.55% are self-employed, and 40.44% did not have a family member infected with COVID-19. The risk perception of COVID-19 infection was associated with participants’ family antecedent of COVID-19 (p < 0.05). Regarding the factors analyzed, perceived susceptibility to COVID-19 was associated with age (p=0.002), occupation (p<0.05), and a history of COVID-19 (p<0.05), while the perceived benefits of adopting preventive measures against this disease were associated with educational level (p < 0.001).

**Conclusions:** The risk perception of contracting COVID-19 was higher among whose had multiple infected relatives.. Furthermore, the perception of susceptibility and the perceived benefits of using preventive measures were associated with sociodemographic characteristics.

## Introduction

Emerging viral infections pose a threat to public health due to their rapid transmission and the increased morbidity and mortality rates in the population. At the end of the coronavirus disease 2019 (COVID-19) pandemic worldwide, over 192 million people were reported to have been infected, with 9.2 million deaths resulting from this viral disease. ^1^

To prevent the spread of COVID-19, the World Health Organization (WHO) recommended pharmacological measures (vaccines and antiretroviral drugs) and non-pharmacological measures such as hand hygiene, mask usage, and physical distancing. At the beginning of the pandemic, studies conducted in different countries reported that the population had a high level of knowledge, favorable attitudes, and appropriate practices regarding these measures. ^2^ However, subsequent research reported that although the population had a good level of knowledge, adherence to preventive measures was low. ^3^

Based on the theory of common-sense model of self-regulation, the decrease in the adoption of prevention measures could be attributed to a change in risk perception. ^4^ Additionally, at the onset of the pandemic, media and government messages increased the population’s perception of risk, leading individuals to adopt preventive measures. However, after this initial stage and with the prolonged presence of the virus, people gradually ceased to fear it and began to perceive it as part of their daily lives. This can be explained by the “mere exposure effect” proposed by Zajonc, ^5^ who suggested that repeated exposure to a stimulus is sufficient to develop a more positive attitude towards that stimulus. Zajonc ^5^ proposed that the relationship between exposure and liking follows a positive deceleration curve, where the initial exposures have a greater impact on the attitude towards the stimulus compared to subsequent exposures.

The strength of the mere exposure effect depends on the individual’s pre-existing attitude towards the stimulus and tends to be stronger when the individual is unaware of the stimulus presented. To support this hypothesis, Zajonc discussed three types of supporting studies: word frequency and word evaluation, interpersonal contact and interpersonal attraction, and familiarity with musical selections and other stimuli for which individuals express their liking. ^6^ In other words, when the brain encounters a new or unfamiliar stimulus, it activates the amygdala (fear response). However, with repeated exposure to that stimulus, familiarity is generated, leading to increased confidence. In other words, living with a risk factor makes the brain perceive it as more familiar, gradually decreasing the perception of risk.

The risk perception is a relevant topic in scientific research, and it has become a multidisciplinary field which includes several disciplines, such as psychology, sociology, economics, and communication science. The models developed to understand the risk perception have progressed significantly in the last decades, as long as the empiric evidences have been accumulated.

In the context of psychology, the risk perception is defined as “the subjective evaluation made by an individual about the probability and the impact of future negative consequences”. ^6^ This definition highlights the subjective nature of risk perception, which varies from one individual to another, and it can be influenced by cognitive, emotional, and social factors.

One of the most influential and pioneering models in the study of the risk perception is the Psychometric Paradigm proposed by Slovic. ^6^ This model is based on the idea that the risk perception is influenced by two main dimensions: dread risk and unknown risk. Slovic argues that the risks which create fear and are perceived as unknown tend to be considered as the most dangerous ones by the population.

Other important historic approach is the Risk Amplification Model developed by Kasperson et al. ^7^ This model focuses in how social, cultural, and political factors can increase or mitigate the risk perception in a community. The authors argue that the risk perception is a social construction and that communities can increase the risk perception through media communication and social interaction.

In the field of risk communication, the Strategic Risk Communication Model of Covello et al. ^8^ was fundamental. This model focuses on how organizations and communicators can address the risk perception through effective communication strategies. This is based on the idea that the risk perception is influenced by factors, such as confidence in sources of information and transparency in communication.

The relationship between the risk perception and the Health Belief Model (HBM) is reinforced by the idea that the risk perception is a key component of the health beliefs. The risk perception influences the perception of susceptibility and severity, two main factors of HBM.

The risk perception can also act as a ‘signal’ that activates preventive decision making. The relationship between the risk perception and the Health Belief Model is fundamental to understand how individual beliefs and perceptions affect the adoption of preventive health behaviors. The risk perception acts as a key component inside the HBM, influencing the perception of susceptibility and severity, and being used as a motivational factor for the preventive decision making.

Although several studies highlight the association between adherence to COVID-19 prevention measures and risk perception, ^9^^,^^10^ few studies analyze the risk perception of coronavirus infection. A study conducted in Brazil that developed a scale on risk perception based on the Health Belief Model (HBM) indicates that factors such as the type of transportation used, low-income individuals, and those with autoimmune diseases significantly affect risk perception. ^11^ Furthermore, in addition to stay-at-home measures, different non-uniform patterns of behavior in the population have been demonstrated. Specifically, demographic, socioeconomic, and environmental density characteristics were associated with health outcomes related to exposure risk. ^12^

Analyzing the factors associated with the perception of contagion risk is an important psychological contribution to understanding population behavior during this and future pandemics. In addition, having studies that explore risk perception in the Peruvian population will allow the design of proposals to reduce risky behaviors and promote the adoption of preventive measures in future pandemics. Therefore, this research aimed to determine the association between the risk perception of contracting COVID-19 and sociodemographic characteristics among Peruvian population.

## Methods

### Participants

The population consisted of residents from four cities in Peru (Callao, La Libertad, Lambayeque, and Lima Metropolitana). The study included residents of the selected cities who had access to an internet-connected device. Participants under 18 years old and those who were unavailable to complete the questionnaire were excluded. The sample size was determined in 492 participants considering that it was expected to find moderate to small size effects (f = 0.15) and a statistical power of 80%. However, data was collected from 1044 participants due to the fact possibility of having significant losses in data due to non-complete answers and also to increase the statistical power. Participants taking part in the online survey were chosen using a convenience sampling method.

### Study design and instruments

This was an analytical and cross-sectional study in which participants were surveyed using a self-administered questionnaire developed in Google Forms. ^31^ The questionnaire was distributed via email and social media from October to December, 2021.

The online form consisted of three sections. The first section included the informed consent. The second section collected information on sociodemographic characteristics (sex, age in terms of the complete years from your birthday, marital status, highest level of education, occupation, personal and family history of COVID-19 in the household). The third section had the scale of risk perception of contracting the COVID-19 infection, which was designed based on the Health Belief Model (HBM) and validated in Brazil. ^11^ The scale had 24 questions that assessed five factors: a) perceived susceptibility, corresponding to knowledge and belief about the possibility of getting coronavirus; b) perceived severity, asking about personal beliefs about how the individual would experience the disease process and the intensity of symptoms; c) perceived benefits, corresponding to the effectiveness of adopted behavioral mechanisms to prevent infection; d) perceived barriers, aiming to understand the difficulties in adhering to protective and preventive measures for coronavirus transmission, and e) health motivation, seeking to improve overall health. Responses were rated on an analog scale from 0 to 100, where zero represented “not at all” and 100 represented “extremely high.”

Before the validation process, a back-translation was performed. Firstly, the questionnaire was translated from Portuguese to Spanish by a certified translator, and then vice versa. Subsequently, a pilot test was conducted with 30 participants to evaluate the comprehension of the questions and response options. In this activity, it was identified that participants had difficulty rating the probability of risk (response option) with a value greater than 100. This is why the range of response options was modified from the original version, which ranged from 0 to 120, to a range from 0 to 100.

The content of the instrument went through a process of expert judgment validation. In this process, health professionals who are methodologists, members of the COVID-19 command, and epidemiologists.

In order to explore the reliability of the instrument, a split-half correlation procedure was conducted, correlating all possible halves of items. Based on the obtained correlations, a data distribution was obtained for each subscale of the instrument, and the 2.5th, 50th, and 97.5th percentiles are reported. Split-half correlations with a score of 0.70 or higher are considered adequate.

### Reliability analysis

Table 1 shows the results of the reliability analysis conducted for the scales of perception of COVID-19 contagion risk and its factors. Specifically, we can observe that the median split-half correlations for the general risk perception scale (0.91), susceptibility scale (0.83), and severity scale (0.88) are considerably higher than 0.70, indicating that these scales have very good levels of reliability. On the other hand, the severity, benefit, and barrier scales have low levels of reliability as the median split-half correlations are below 0.70 (0.67, 0.53, 0.57, respectively). Regarding the descriptive statistics of the subscales, it can be observed that the highest average perception of risk is found in the barrier subscale (M = 42.34, SD = 18.03), while the lowest average perception of risk is observed in the benefit perception (M = 28.90, SD = 15.68), followed by susceptibility perception (M = 28.94, SD = 19.50).

**Table 1.** Quantiles of split-half correlation reliability analysis and descriptive statistics of the COVID-19 risk perception scale.

	Quantiles r			M	SD	Min	Max
	2.5	50	97.50				
General risk perception	0.85	0.91	0.93	34.88	14.05	1.17	80.83
Susceptibility	0.80	0.83	0.85	28.94	19.50	0	100
Severity	0.80	0.88	0.89	36.18	21.12	0	100
Benefit	0.59	0.67	0.68	28.90	15.68	0	94.00
Barrier	0.42	0.53	0.57	42.34	18.03	1.40	100
Motivation	0.51	0.57	0.65	36.61	16.52	0	100

### Statistical analysis

To ascertain the differences in susceptibility to COVID-19 based on sex, marital status, education level, occupation, and family history, a combination of Student’s t-tests and Analysis of Variance (ANOVA) was utilized, contingent on the number of categories within the variable under consideration. When statistically significant disparities were identified using ANOVA, a post hoc Tukey analysis was implemented to elucidate the nature of these differences. Consistently, the assumptions of parametric analyses were validated by constructing half-normal simulation plots for each model to assess the distribution of residuals and homoscedasticity. As indicated by Moral et al., ^13^ Conventional tests for normality and homoscedasticity, such as Shapiro-Wilk and Bartlett, can be sensitive to large sample sizes. A more robust method to verify the adherence to these assumptions is to construct plots that juxtapose residuals with a half-normal simulation, while also illustrating 95% confidence bands to determine if the observed distribution aligns with outcomes where the assumptions are satisfied. Visual representations of these analyses can be viewed in Supplemental Material, confirming that in all instances the assumptions of parametric analysis were met. Lastly, to investigate the correlation between age and susceptibility to COVID-19, a Spearman correlation coefficient was computed, given that age did not adhere to a normal distribution. All analyses were executed using R software v4.2.1. ^14^

### Potential biases

Due to the absence of an available sampling frame and the utilization of convenience sampling, the sample obtained in this study was not representative of the population across the four Peruvian cities, limiting the ability to generalize the findings. Nonetheless, the number of participants in this study, nearly twice the minimum sample size, allows for statistically robust results. On another note, as the survey was conducted virtually, the results may be subjected to biases, such as social desirability bias—the tendency for respondents to provide socially acceptable responses rather than responding truthfully.

Lastly, given that the participants had to respond numerically to the risk perception questionnaire, potential difficulty in providing responses may have arisen, as some individuals have limitations in quantitatively evaluating their perceptions, or confusion surrounding the interpretation of the questions. For this reason, clear instructions were provided in the questionnaire, and each question reiterated the numerical scale that could be used for responses. In addition, rigorous quality control was conducted on the responses to select the questionnaires that were considered for statistical analysis.

### Ethical considerations

The study was approved on May 24, 2021 by the Institutional Ethics Committee of Norbert Wiener University (file number 560-2021). All participants provided informed consent before responding to the questionnaire. The data collected through the survey in each city were coded, excluding identifying information, ensuring the confidentiality of the information and exclusive access to the data by the researchers.

## Results

### Sociodemographic data

A total of 1044 participants answered, of which 223 were excluded for quality control purposes because they provided letters or words instead of indicating a number between 0 and 100 in their responses. ^33^ Out of the 821 participants, 53.71% (n = 441) were women, with an average age of 28.29 years (SD = 11.56). Regarding marital status, 76.49% (n = 628) reported being single, 20.95% (n = 172) reported being married, while 2.56% (n = 21) reported being separated or divorced. In terms of participants’ education level, it was observed that 73.33% (n = 602) had a higher education level, while 26.31% (n = 216) had completed secondary education. In regard to the occupation of the participants, 45.55% (n = 374) reported being self-employed, 42.39% (n = 348) reported being employed, 11.33% (n = 93) reported being homemakers, and the remaining 0.73% (n = 6) indicated being retired. Finally, with regard to the history of COVID-19 among family members or individuals living in the same household, at the time of the survey, 40.44% (n = 332) of the participants reported that no family member or household member had contracted COVID-19, 28.01% (n = 230) reported that one family member had been infected, while the remaining 31.55% (n = 259) reported that several members of their family and household had been infected with COVID-19.

In Table 2, the Pearson correlations for the different scales of risk perception can be observed. In this table, it can be seen that the general scale of risk perception maintains a high and significant correlation with all the subscales (0.72 < r’s < 0.80). On the other hand, the different subscales show moderate to strong correlations if (0.32 < r’s < 0.60).

**Table 2.** Correlation matrix of risk perception scales.

	1	2	3	4	5
1. General risk perception of contracting COVID-19	-				
2. Susceptibility	0.74 *	-			
3. Severity	0.76 *	0.55 *	-		
4. Benefit	0.80 *	0.50 *	0.48 *	-	
5. Barrier	0.72 *	0.32 *	0.38 *	0.57 *	-
6. Motivation	0.79 *	0.54 *	0.48 *	0.60 *	0.54 *

* p < 0.001.

### Analysis of risk perception of contracting COVID-19

In Table 3, the Student’s t-tests for the means of risk perception of contracting the COVID-19 infection can be observed according to the reported sex of the participants. Specifically, it can be indicated that none of the subscales showed statistically significant differences (p’s > 0.5). These results indicate that the risk perception of contracting the COVID-19 infection is the same for men and women.

**Table 3.** Comparison of means of risk perception of contracting the COVID-19 infection according to the participants’ sex.

	Men		Women		t
	M	SD	M	SD	
General risk perception	35.01	14.12	34.72	13.99	0.3
Susceptibility	28.49	19.90	29.46	19.03	-0.71
Severity	36.63	20.63	35.65	21.70	0.66
Benefit	29.09	15.78	28.69	15.57	0.36
Barrier	42.27	17.58	42.42	18.56	-0.11
Motivation	37.23	16.62	35.89	16.41	1.16

In Table 4, the Analysis of Variance (ANOVA) comparisons of the means of risk perception of contracting the COVID-19 infection can be observed according to the marital status reported by the participants. Particularly, it can be observed that none of the subscales showed statistically significant differences (p’s > 0.5). These results indicate that the risk perception of contracting the COVID-19 infection is the same for married, single, and separated participants.

**Table 4.** Comparison of means of risk perception of contracting the COVID-19 infection according to the participants’ marital status.

	Married		Separated		Single		F
	M	SD	M	SD	M	SD	
General risk perception	35.7	15.16	39.37	15.24	34.5	13.68	1.59
Susceptibility	31.2	21.10	32.93	20.43	28.18	18.97	2.08
Severity	37.32	22.79	39.6	15.83	35.75	20.80	0.66
Benefit	27.85	15.22	33.98	20.97	29.02	15.59	1.51
Barrier	43.92	18.33	46.83	18.64	41.75	17.91	1.65
Motivation	37.09	17.03	43.11	20.30	36.26	16.23	1.84

In Table 5, we can observe the results of the Analysis of Variance (ANOVA) for the means of risk perception of contracting the COVID-19 infection according to the participants’ educational level. For this sociodemographic variable, significant differences were found in the benefit subscale based on educational level (F = 5.75, df = 2,818, p = 0.003). According to the post-hoc Tukey analysis, participants with a secondary education level have a significantly higher benefit score than those with a higher education level (p = 0.004). No other significant differences were observed in any of the other subscales of risk perception of contracting the COVID-19 infection.

**Table 5.** Comparison of means of risk perception of contracting the COVID-19 infection according to the participants’ educational level.

	Primary		Secondary		Higher		F
	M	SD	M	SD	M	SD	
General risk perception	43.00	12.03	36.18	14.28	34.37	13.95	1.84
Susceptibility	34.00	20.3	28.48	18.47	29.07	19.88	0.17
Severity	56.47	7.31	37.83	20.95	35.49	21.16	2.38
Benefit	39.00 _ab_	10.67	31.78 _a_	17.88	27.82 _b_	14.7	5.75 **
Barrier	50.33	13.62	42.93	17.7	42.09	18.17	0.47
Motivation	30.75	9.93	37.98	15.98	36.15	16.73	1.17

Note: The subscripts with different letters indicate significant differences according to the post-hoc Tukey’s test.

* p < 0.05.

** p < 0.01.

*** p < 0.001.

In Table 6, we can see the results of the Analysis of Variance (ANOVA) for the mean scores of risk perception of contracting the COVID-19 infection according to participants’ occupation. For this variable, significant differences were observed in the susceptibility subscale (F = 7.47, df = 3,817, p < 0.001). According to the post-hoc Tukey analysis, participants who are employed reported higher levels of susceptibility compared to homemakers (p = 0.008). Similarly, participants who are employed reported higher levels of susceptibility compared to self-employed participants (p < 0.001). No significant differences were found in the other subscales of the instrument.

**Table 6.** Comparison of mean scores of risk perception of contracting the COVID-19 infection according to participants’ occupation.

	Homemaker		Employed		Self-employed		Retiree		F
	M	SD	M	SD	M	SD	M	SD	
General risk perception	33.46	14.56	33.76	14.12	34.37	13.79	37.14	18.01	0.98
Susceptibility	25.49 _a_	18.36	32.63 _b_	20.43	26.36 _a_	18.32	28.57 _ab_	21.86	7.47 ***
Severity	36.11	21.48	35.47	21.00	36.79	21.14	40.03	25.01	0.3
Benefit	28.17	17.42	28.82	15.06	29.24	15.84	24.43	14.98	0.29
Barrier	39.05	18.42	42.92	17.37	42.38	18.31	56.93	25.24	2.48
Motivation	37.34	15.00	37.79	17.51	35.44	15.91	29.5	14.55	1.67

Note: The subscripts with different letters indicate significant differences according to the post-hoc Tukey’s test.

* p < 0.05.

** p < 0.01.

*** p < 0.001.

Table 7 shows the results of the ANOVA for the mean scores of risk perception of contracting the COVID-19 infection according to participants’ family history of COVID-19. Significant differences were observed in the general perceived risk scale (F = 3.06, df = 2,818, p = 0.047) and the susceptibility scale (F = 9.66, df = 2,818, p < 0.001) for this sociodemographic variable. Following post-hoc Tukey analysis, it was found that participants with multiple infected family members had higher levels of perceived risk on the general perceived risk scale compared to participants with no infected family members (p = 0.04). In terms of the susceptibility scale, participants with no infected family members reported lower levels of susceptibility compared to those with at least one infected family member (p = 0.03), and those with multiple infected family members (p < 0.001). No other significant differences were observed in any of the other subscales of risk perception of contracting the COVID-19 infection.

**Table 7.** Comparison of mean scores of risk perception of contracting the COVID-19 infection according to participants’ family history of COVID-19.

	No		If one		If more than one		F
	M	SD	M	SD	M	SD	
General risk perception	33.56 _a_	13.86	35.03 _ab_	13.62	35.43 _b_	14.54	3.06 *
Susceptibility	25.59 _a_	18.53	29.73 _b_	19.72	32.52 _b_	19.87	9.66 ***
Severity	35.44	21.47	35.79	20.09	37.47	21.58	0.73
Benefit	27.64	15.38	30.20	15.65	29.38	16.02	1.99
Barrier	42.33	18.55	41.60	17.27	43.00	18.05	0.37
Motivation	35.43	16.40	36.07	16.76	38.59	16.35	2.84

Note: The subscripts with different letters indicate significant differences according to the post-hoc Tukey’s test.

* p < 0.05.

** p < 0.01.

*** p < 0.001.

Finally, regarding the correlation between age and perceived risk, Table 8 reveals that only the susceptibility scale shows a positive, weak, and significant relationship with age (Rho = 0.11, p = 0.002). This indicates that older participants in the sample reported higher susceptibility scores compared to younger participants. None of the other subscales showed significant associations.

**Table 8.** Spearman correlations between risk perception of contracting the COVID-19 infection and participants’ age.

	Age	
	Rho	p
General risk perception	0.03	0.44
Susceptibility	0.11	0.002
Severity	0.02	0.62
Benefit	-0.06	0.11
Barrier	0.03	0.33
Motivation	0.01	0.85

## Discussion

In this study, after validating a scale of risk perception of contracting the COVID-19 infection perceived in the Peruvian population, which was constructed based on the HBM, differences in perception (global scale and two factors) were described according to certain sociodemographic characteristics. This finding contributes to the growing literature on addressing risk perception of contracting the COVID-19 infection based on the HBM to understand its variability in the population.

The direct and positive correlation between age and perceived susceptibility to COVID-19 found in this study suggests that as individuals get older, they have more favorable beliefs regarding the likelihood of contracting coronavirus. Considering that studies comparing different age groups have found that the adoption of preventive behaviors is generally associated primarily with susceptibility and to a lesser extent with the perception of the impact of COVID-19, this finding is particularly interesting. It implies that older adults, with a higher perception of susceptibility, are more likely to adopt preventive measures. On the other hand, younger people having less favorable perceptions regarding the probability of getting infected could represent a risk group in terms of adopting preventive behaviors against COVID-19. Therefore, preventive interventions should be targeted towards these population groups.

Similarly, a study conducted during the first wave of infections in Argentina found that perceived severity was positively associated with age. ^15^ Additionally, another study conducted in Italy found that the perception of susceptibility among older adults was higher compared to younger individuals. ^16^ Conversely, a study that used the HBM in Canada and analyzed differences in the perception of personal impact of COVID-19 across age groups found that older adults, compared to younger individuals, had greater concerns about being hospitalized or dying (severity), but not about the risk of infection despite having higher susceptibility. ^17^ Furthermore, another study conducted in the United States showed that older individuals perceive themselves as less susceptible to getting sick, but are more likely to experience severe consequences if they do contract the disease. ^18^ Even though the literature has reported an association between age and different perceptions of risk related to COVID-19, ^19^^–^^21^ the contribution of this study confirms that susceptibility to the possibility of contracting COVID-19 increases with age.

Additionally, differences in perceived susceptibility to COVID-19 were found based on occupation. Specifically, employed individuals had higher mean scores compared to retirees and homemakers. This finding is consistent with the results of a study conducted in Iran, where they reported that the mean score of risk perception of contracting the COVID-19 infection among employed individuals was up to seven times lower than that of homemakers and retirees. ^22^

As observed in all the presented tables, the mean values related to perceived barriers are higher than those of the other dimensions. This result can be interpreted as a general difficulty for study participants in finding behaviors and actions in the face of the possibility of contamination. Possible income and salary reductions, fear of job loss, and limited transportation alternatives without crowds could be examples of events contributing to greater resistance to adopting behaviors that reduce exposure to the virus. Furthermore, the weak to moderate correlation between perceived barriers and perceptions of benefits and susceptibility indicates that even when individuals are aware of the benefits and conditions that make them more susceptible, the lack of options to modify their work and living conditions is evident in the population.

Additionally, it was found that participants who had multiple previous COVID-19 infections had a higher perceived susceptibility score for COVID-19 compared to those with a single infection or those who reported not being infected with the virus. Similarly, the risk perception of university students in China whose family members or friends had been exposed to confirmed or suspected COVID-19 patients was higher than those who had not been exposed. ^23^ Furthermore, another study conducted in ten countries reported that direct experience is a predictor of COVID-19 risk perception. ^11^ One possible reason could be that awareness of the ineffectiveness of the immune system in preventing the disease and the experience of being diagnosed with COVID-19 lead to an increased susceptibility perception of COVID-19.

On the other hand, regarding the perception of benefits, the sociodemographic characteristic in which differences were found was the level of education. Specifically, individuals with primary education perceive greater efficacy of the mechanisms adopted to prevent COVID-19 infection compared to those who completed secondary or higher education. This could be explained by research conducted in Peru, which reported that a higher level of education among household heads was associated with higher scores of myths and inappropriate beliefs. It is worth noting that in that study, the most frequent myths were “spraying alcohol or chlorine kills the virus” and “home remedies can cure or prevent coronavirus”. ^24^ However, in a study conducted in four cities in Latin America, older adults with higher academic degrees showed better adherence to self-care measures. ^25^

Regarding the perception of risky behaviors for COVID-19 transmission, it was found that participants who had multiple infected family members had higher scores than those who had no infected family members. One explanation for this finding could be that people have a higher perception of risk for COVID-19 when they perceive themselves as more personally vulnerable to infection or when they perceive the pandemic as more severe. ^26^

When comparing the mean perception scores in each of the dimensions obtained in this study conducted in the Peruvian population, it can be observed that in four out of the five dimensions, the score is lower compared to the study conducted in Brazil at the beginning of the pandemic. ^12^ This indicates that as the population becomes more familiar with the coronavirus, due to the mere exposure effect, their perception of risk decreases. ^27^ Additionally, the decrease of the risk perception could be explained because as of October 2021, in the Americas Region, it was reported a decrease in the infection rates and COVID deaths, and the beginning of COVID-19 vaccination generated rejection and uncertainty ^28^ because the population perceived a lower susceptibility risk and therefore had less motivation to adopt other preventive measures.

### Implications

Overall, the findings of this study, in line with the literature, highlight the impact of sociodemographic variables on susceptibility perception and perception of benefits. This aspect can be useful in targeting public health interventions aimed at calibrating risk perception in the population to promote compliance with preventive measures, as preventive behavior is only evident when the event is perceived as highly contagious or dangerous. ^26^ While risk perception influences the adoption of preventive behaviors during a pandemic, ^23^^,^^29^^,^^30^ especially the affective dimension, this construct alone is not sufficient to promote the adoption of these behaviors. ^27^ Therefore, to reduce the incidence of the disease, it is necessary to consider the sociodemographic characteristics of the population, complemented by the issuance of prevention guidelines, and thus ensure adequate vaccination coverage. ^31^^,^^32^

Another implication of this study stems from the psychometric analysis conducted, which demonstrated that the scale developed in Brazil to measure the perception of risk behaviors for COVID-19 contagion based on the health belief model ^12^ exhibits adequate psychometric properties in the Peruvian population. This suggests that this scale could be used in other Latin American countries with a previous a validation process.

### Limitations

The first limitation is that data collection was based on self-reporting, which could increase the likelihood of common method variance. However, these results represent an initial approach to the evaluated phenomenon and should be verified using complementary methodologies to control this effect. The second limitation is related to the convenience sampling method employed, which allowed quick data collection and access to remote areas of Peru during the pandemic but diminished the representativeness of the sample. The third limitation is the unequal composition of the sample according to sociodemographic characteristics, which could impact the detection of differences. Nevertheless, the statistical tests employed enabled the identification of statistically significant differences. Despite these limitations, this study reports findings from residents of different cities in Peru.

## Conclusion

This study found that perceived susceptibility to COVID-19 correlates with age, occupation, and having a history of COVID-19 infection. The perceived benefits of adopting preventive measures for this disease are associated with educational level. Furthermore, the perception of risk of coronavirus contagion is linked to the history of infection with this viral disease. Additionally, the psychometric properties of a scale measuring the perception of risk behaviors related to COVID-19 in the Peruvian population, developed under the HBM, have been confirmed.

## Data Availability

Figshare: Risk perception dataset English.xlsx. https://doi.org/10.6084/m9.figshare.23703939.v1. ^33^ Figshare: Questionnaire on risk perception of contracting COVID-19 in a Peruvian population. https://doi.org/10.6084/m9.figshare.23669154.v1. ^34^ Data are available under the terms of the Creative Commons Attribution 4.0 International license (CC-BY 4.0).
