# Supplementary material for: Association between the risk perception of contracting COVID-19 and sociodemographic characteristics in a Peruvian population
Source: F1000Res. 2023 Oct 30;12:919. Originally published 2023 Aug 1. [Version 2] doi: 10.12688/f1000research.138838.2 (PMC10804047; doi:10.12688/f1000research.138838.2)
Supplement: Peer review history

# Reviewer report: Reviewer response for version 2

**DOI:** 10.5256/f1000research.158078.r231115

**Published:** 2024-01-22

**Authors:** Mahmoud Abdel Hameed Shahin

**Affiliations:** 1 Prince Sultan Military College of Health Sciences, Dhahran, Saudi Arabia

First and foremost, I would like to express my gratitude for the opportunity to review this noteworthy paper intended for publication in this esteemed journal.

 The research topic under consideration holds significant importance for public health, particularly in the context of the ongoing COVID-19 pandemic. The paper is commendably well-structured, with lucid ideas and a concise, argumentative writing style. Nevertheless, I have a few minor comments that warrant attention:

1. I recommend including a table that outlines the sociodemographic characteristics of the participants. This addition would enhance the clarity and comprehensiveness of the presented data.
2. It would be beneficial to elaborate on the risk perception levels and their interpretation based on cut scores within the sample of the Peruvian population. A brief discussion on this aspect would contribute to a more thorough understanding of the research findings.
3. In the analysis of results presented in Table 3, the statement "These results indicate that the risk perception of contracting the COVID-19 infection is the same for men and women" may lead to misinterpretation. To rectify this, I suggest using a more precise expression, such as "yielded relatively similar results without a statistically significant difference." This adjustment will accurately convey the findings.
4. Consider incorporating relevant and recent studies into the discussion of your study findings. For instance:

- Shahin and Hussien (2020 ^1^).
- Cipolletta *et al.* (2022 ^2^).

Is the work clearly and accurately presented and does it cite the current literature?

Yes

If applicable, is the statistical analysis and its interpretation appropriate?

Partly

Are all the source data underlying the results available to ensure full reproducibility?

Yes

Is the study design appropriate and is the work technically sound?

Yes

Are the conclusions drawn adequately supported by the results?

Yes

Are sufficient details of methods and analysis provided to allow replication by others?

Yes

Reviewer Expertise:

Critical care nursing, infectious diseases, medical surgical nursing

I confirm that I have read this submission and believe that I have an appropriate level of expertise to confirm that it is of an acceptable scientific standard, however I have significant reservations, as outlined above.

## References

[1] : Risk perception regarding the COVID-19 outbreak among the general population: a comparative Middle East survey. *Middle East Current Psychiatry* .2020;27(1) : 10.1186/s43045-020-00080-7 10.1186/s43045-020-00080-7

[2] : Risk Perception towards COVID-19: A Systematic Review and Qualitative Synthesis. *Int J Environ Res Public Health* .2022;19(8) : 10.3390/ijerph19084649 10.3390/ijerph19084649 35457521 PMC9028425

# Reviewer report: Reviewer response for version 2

**DOI:** 10.5256/f1000research.158078.r219331

**Published:** 2023-11-21

**Authors:** María de la Concepción Pérez de Celis-Herrero

**Affiliations:** 1 Facultad de Ciencias de la Computación, Laboratorio de Tecnologías para la Salud, Benemerita Universidad Autonoma de Puebla, Puebla, Puebla, Mexico

I consider that the authors made 95% of the recommendations but they have not yet cited the HBM questionnaire, differences in perception (global scale and two factors), adapted for this study and I believe that it is important that they cite it correctly. On the other hand, I think it is advisable to include the questionnaire used in an annex.

Is the work clearly and accurately presented and does it cite the current literature?

Yes

If applicable, is the statistical analysis and its interpretation appropriate?

I cannot comment. A qualified statistician is required.

Are all the source data underlying the results available to ensure full reproducibility?

No source data required

Is the study design appropriate and is the work technically sound?

Partly

Are the conclusions drawn adequately supported by the results?

Partly

Are sufficient details of methods and analysis provided to allow replication by others?

Partly

Reviewer Expertise:

Public Health and Computer Science

I confirm that I have read this submission and believe that I have an appropriate level of expertise to confirm that it is of an acceptable scientific standard.

# Reviewer report: Reviewer response for version 1

**DOI:** 10.5256/f1000research.152068.r193525

**Published:** 2023-09-08

**Authors:** Evelyn Fernández-Castillo

**Affiliations:** 1 Psychology Department, Universidad Central Marta Abreu de las Villas, Santa Clara, Villa Clara, Cuba

The manuscript approaches a current topic. The results presented contribute to refining health interventions for future emergency situations.

 It is considered that the introduction should: 

- Deepen the definition of risk perception addressed in the research. 
- Highlight the relationship between the definition assumed and the Health Belief Model and its importance for this research.

In the methodological section and analysis of results: 

- It is stated that its uses a non-probabilistic sampling by convenience. And in the description of the statistical analyses performed, it is stated that "In all cases, the assumptions of parametric analyses were verified", however, in the results section, the results that allow verifying the fulfillment of these assumptions are not made explicit. I suggest incorporating this into the manuscript.

Is the work clearly and accurately presented and does it cite the current literature?

Partly

If applicable, is the statistical analysis and its interpretation appropriate?

Partly

Are all the source data underlying the results available to ensure full reproducibility?

Yes

Is the study design appropriate and is the work technically sound?

Yes

Are the conclusions drawn adequately supported by the results?

Yes

Are sufficient details of methods and analysis provided to allow replication by others?

Yes

Reviewer Expertise:

Psychology, addiction prevention, mental health, disease prevention, health promotion, validation of psychological evaluation instruments

I confirm that I have read this submission and believe that I have an appropriate level of expertise to confirm that it is of an acceptable scientific standard, however I have significant reservations, as outlined above.

# Reviewer report: Reviewer response for version 1

**DOI:** 10.5256/f1000research.152068.r193526

**Published:** 2023-08-22

**Authors:** María de la Concepción Pérez de Celis-Herrero

**Affiliations:** 1 Facultad de Ciencias de la Computación, Laboratorio de Tecnologías para la Salud, Benemerita Universidad Autonoma de Puebla, Puebla, Puebla, Mexico

This article analyzes the perception of the risk of contracting COVID-19 in Peru taking into account the socioeconomic characteristics of the respondents. For the authors, the analysis of the factors associated with the perception of contagion risk is an important psychological contribution to understanding the behavior of the population during this and future pandemics and will allow the design of proposals to reduce risk behaviors and promote the adoption of preventive measures in the Peruvian population.

 To carry out the study, an online questionnaire of our own design with 24 questions based on the Health Belief Model (HBM) was used, but this questionnaire is not included in the article, only the five factors that were evaluated are mentioned: a) perceived susceptibility, corresponding to knowledge and belief about the possibility of contracting coronavirus; b) perceived severity, asking about personal beliefs about how the individual would experience the disease process and the intensity of the symptoms; c) perceived benefits, corresponding to the effectiveness of the behavioral mechanisms adopted to prevent infection; d) perceived barriers, with the aim of understanding the difficulties in adhering to coronavirus transmission protection and prevention measures, and e) health motivation, seeking to improve overall health. Reason why I consider that this study cannot be reproduced by other working groups. The questionnaire was applied in four cities in Peru from October to December 2021. The sample consisted of 821 people aged 18 and over.

 The authors conclude that perceived susceptibility to COVID-19 is correlated with age, occupation, and having a history of COVID-19 infection. The perceived benefits of adopting preventive measures for this disease are associated with the educational level. In addition, the perception of risk of contagion from coronavirus is linked to a history of infection by this viral disease.

 It should be noted that at the time the study was carried out in Peru, vaccination against covid19 had already begun (start date February 9, 2021), starting with health personnel, older and vulnerable groups, continuing with the rest of the groups. of age to reach most of the target population of 18 years and over during the year 2021[1]. And if we add to this that in October 2021 PAHO[2] reported that the rates of infection of COVID-19 in the region of the Americas was at the lowest levels in 2021, we can assume that the results found could be influenced by both facts, which is why I would assume that the conclusions are partially supported by the results.

Is the work clearly and accurately presented and does it cite the current literature?

Yes

If applicable, is the statistical analysis and its interpretation appropriate?

I cannot comment. A qualified statistician is required.

Are all the source data underlying the results available to ensure full reproducibility?

No source data required

Is the study design appropriate and is the work technically sound?

Partly

Are the conclusions drawn adequately supported by the results?

Partly

Are sufficient details of methods and analysis provided to allow replication by others?

Partly

Reviewer Expertise:

Public Health and Computer Science

I confirm that I have read this submission and believe that I have an appropriate level of expertise to confirm that it is of an acceptable scientific standard, however I have significant reservations, as outlined above.

## References

[1] : Efectividad de vacunas COVID-19 y riesgo de mortalidad en Perú: Un estudio poblacional de cohortes pareadas. *Anales de la Facultad de Medicina* .2022;83(2) : 10.15381/anales.v83i2.21531 87-94 10.15381/anales.v83i2.21531

[2] La región de las Américas ha alcanzado las tasas más bajas de COVID-19 en el último año. Reference source
